# Assessment of selection bias in a health survey of children and families – the IDEFICS Sweden-study

**DOI:** 10.1186/1471-2458-13-418

**Published:** 2013-05-01

**Authors:** Susann Regber, Masuma Novak, Gabriele Eiben, Lauren Lissner, Sabrina Hense, Tatiana Zverkova Sandström, Wolfgang Ahrens, Staffan Mårild

**Affiliations:** 1 Nordic School of Public Health NHV, Box 12 133, SE- 402 42, Gothenburg, Sweden; 2 Department of Molecular and Clinical Medicine, the Sahlgrenska Academy, University of Gothenburg, Gothenburg, Sweden; 3 Department of Public Health and Community Medicine, Public Health Epidemiology Unit Sahlgrenska Academy, University of Gothenburg, Gothenburg, Sweden; 4 Department of Epidemiological Methods and Etiologic Research, Institute for Epidemiology and Prevention Research (BIPS), Bremen, Germany; 5 Department of Paediatrics, Institute of Clinical Sciences, Sahlgrenska Academy, University of Gothenburg, Gothenburg, Sweden

**Keywords:** Selection bias, Children, Obesity, Health survey, Registers

## Abstract

**Background:**

A health survey was performed in 2007–2008 in the IDEFICS/Sweden study (Identification and prevention of dietary- and lifestyle-induced health effects in children and infants) in children aged 2–9 years. We hypothesized that families with disadvantageous socioeconomic and -demographic backgrounds and children with overweight and obesity were underrepresented.

**Methods:**

In a cross-sectional study, we compared Swedish IDEFICS participants (N=1,825) with referent children (N=1,825) using data from Statistics Sweden population registers. IDEFICS participants were matched for age and gender with a referent child living in the same municipality. Longitudinal weight and height data from birth to 8 years was collected for both populations (n=3,650) from the children’s local health services. Outcome measures included the family’s socioeconomic and demographic characteristics, maternal body mass index (BMI) and smoking habits before pregnancy, the children’s BMI standard deviation score (SDS) at the age of inclusion in the IDEFICS study (BMISDS-index), and the children’s BMI-categories during the age-span. Comparisons between groups were done and a multiple logistic regression analysis for the study of determinants of participation in the IDEFICS study was performed.

**Results:**

Compared with IDEFICS participants, referent families were more likely to have lower education and income, foreign backgrounds, be single parents, and have mothers who smoked before pregnancy. Maternal BMI before pregnancy and child’s BMISDS-index did not differ between groups. Comparing the longitudinal data-set, the prevalence of obesity was significantly different at age 8 years n= 45 (4.5%) versus n= 31 (2.9%) in the referent and IDEFICS populations, respectively. In the multivariable adjusted model, the strongest significant association with IDEFICS study participation was parental Swedish background (odds ratio (OR) = 1.91, 95% confidence interval (CI) (1.48–2.47) followed by parents having high education OR 1.80, 95% CI (1.02-3.16) and being married or co-habiting OR 1.75 95% CI (1.38-2.23).

**Conclusion:**

Families with single parenthood, foreign background, low education and income were underrepresented in the IDEFICS Sweden study. BMI at inclusion had no selection effect, but developing obesity was significantly greater among referents.

## Background

In view of the global increase in paediatric obesity [1], the World Health Organization has appealed for public health interventions for prevention [2]. The prevalence of obesity is, however, unevenly distributed by socioeconomic position. Higher rates are seen in countries where income differences are greater, resulting in health inequities [3]. Given the public health priority for preventing childhood obesity and the associated socioeconomic disparities, equity and social justice aspects are considered crucial in health surveys and intervention studies [4,5]. Since body mass index (BMI) is not only a growth measure, but also a more general health determinant, population surveys of BMI might be considered to mirror more general health and equity aspects [5].

In 2006, the IDEFICS study (Identification and prevention of dietary- and lifestyle- induced health effects in children and infants) was launched in eight European countries including Sweden [6]. The aim was to assess children’s health with a focus on overweight and obesity and to develop and evaluate a health-promoting community intervention program. To study children’s health, a survey was performed during the academic year 2007–2008. Whether the participants in this survey were representative of the general population in terms of social and economic condition is, however, unknown. Selection bias might have occurred to some extent, which may introduce bias into the survey findings and conclusions [7,8]. The present study was conducted to assess possible selection bias of participants in the IDEFICS health survey by comparing the socioeconomic, demographic, and anthropometric characteristics of the study population to an unselected reference population.

We hypothesized that 1) families with disadvantageous socioeconomic and demographic backgrounds, and 2) children within the overweight and obesity categories were underrepresented in the IDEFICS health survey in Sweden.

## Methods

### The IDEFICS study

The IDEFICS study was a population-based multi-centre study that included 16,220 children aged 2 to 9 years from eight European countries. Between September 2007 and May 2008, children from schools and kindergartens in selected regions in Italy, Estonia, Cyprus, Belgium, Sweden, Hungary, Germany, and Spain were asked to participate in the baseline study. Municipalities included in IDEFICS were selected to be comparable with regards to sociodemographic and socioeconomic structures within all the countries participating in IDEFICS.

**Figure 1:**
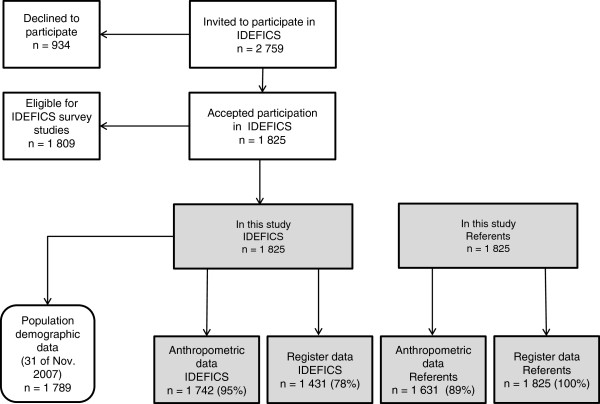
Flow chart for the IDEFICS Sweden register study.

In Sweden, three municipalities in the western part of Sweden participated; Partille, Alingsås, and Mölndal. Schools and kindergartens were used to inform parents about the study [9]. Children’s health was assessed by a thorough physical examination, and the families behavioural and sociodemographic characteristics were investigated, mainly through questionnaires [10]. Of the 2,759 invited children, 1,825 accepted the invitation (Figure 1). These children were eligible for this study. Since a health-promoting community intervention was planned for Partille, it was necessary to recruit half of the IDEFICS participants from there. The inclusion criteria for evaluation in the IDEFICS study was completion of a parental questionnaire and measurement of weight and height of the children (n= 1,809).

### Design

For each of the 1,825 IDEFICS children included in this study, Statistics Sweden [11] selected one referent child from the general population using the unique personal identity numbers (PIN) assigned to all Swedish residents [12]. Each pair was matched with respect to municipality, gender, and age (± 1 month from birth, except for only one child who was extended ± 2 months). By using the list of PIN’s of the IDEFICS and reference populations, the first author (SR) intended to obtain anthropometric data (n = 3,650) from the health records of the children at Child Health Centres (CHC) and School Health Services (SHS) in the three municipalities. After completion, the anthropometric data-set was returned to Statistics Sweden. There, a linkage with data from Statistics Sweden and the Medical Birth Registers (MBR) at the National Board of Health and Welfare was carried out. The PIN´s were then replaced with a serial number. The register data in the data set are valid for the 31 December 2007.

### Data sources

#### Anthropometric data

In Sweden, CHC and SHS are built on voluntary participation and are free of charge. Parents and children attend CHC for health consultations, growth monitoring, and vaccinations. At 2 years of age, 97% had had at least six or more visits [13]. SHS is a continuation of the CHC and is by law [14] offered all children 6–19 years of age. The attendance at SHS is also very high and it is quite unusual to refrain (Renman C., personal communication October 2, 2012).

The EpiData software programme was used [15] at the collection of anthropometric data to transcribe data in a laptop with a remote and safe connection to the Nordic School of Public Health. Anthropometric data was collected for 1,736 (95%) participants in the IDEFICS study population and 1,631 (89%) children in the reference population. Height and weight from birth, 6, 12, and 18 months, and 2.5, 4, 5.5, and 8 years were retrieved and recorded. The time limits to include growth data were ± 2 months for all ages. For children aged 5.5 years, it was extended to ± 0.5 years. For children aged 8 years, the limit was set at ± 1 year.

#### Variables derived from the collected anthropometric data

##### *Body mass index standard deviation score (BMI SDS)*

was determined using the British 1990 referent population [16].

##### *BMI SDS index*

The dates of inclusion of each child in the IDEFICS health survey was used to create the BMI SDS Index. The child’s recorded weights and heights at the CHC and SHS before and after this date were used to calculate this variable as an interpolated BMI SDS.

##### *BMI categories*

by age and gender were defined using BMI cut offs (kg/m^2^) according to International Obesity Task Force [17]*.*We used the categories obesity, overweight, and non-overweight (i.e. normal weight and underweight combined).

#### Register data

Register data were obtained from two Swedish national registers. The first was Statistics Sweden [11], a government agency that produces national statistics and data, e.g. household finances, family demographics, and educational levels. The second was the Medical Birth Register (MBR) at the National Board of Health and Welfare. This is also a national register covering the total population and includes maternal data from pregnancy and delivery and perinatal data of all newborn babies [18]. Register data was retrieved for the 1,431 IDEFICS participants where informed consent had been obtained; 99 parents actively refrained and 295 parents did not respond. In the referent population, register data from all 1,825 families were obtained. No consent was required for these data.

#### Variables from the registers

##### *Disposable income*

The economic standard per consumption unit was defined as the sum of incomes and benefits minus taxes and negative transfers. The sum of incomes, social welfare pension, disability pension, unemployment compensation, and financial study assistance constitute the family’s disposable income. The disposable income was adjusted for family size and dependency burden.

##### *Education*

We used the international educational classification, ISCED 97 [19] which is divided into six levels. Low education is levels 1 and 2, medium is 3 and 4, and high education is 5 and 6. High education includes 2 or more years of education after high school, and low education is ≤ 9 years. The highest education level per household in December 2007 was used in the analysis.

##### *Sociodemographic characteristics*

To standardize the age calculations of the mothers and children, the date of 1 July 2008 was used. The parents of the children were defined as the child’s legal guardians. Thus, the parent(s) could be the biological, foster, or adoptive parent(s). Whether the children were living in families of Swedish or those with a foreign background was defined by the parental country of birth. We classified families as having a Swedish background if one parent was born in Sweden, and foreign if both parents were of non-Swedish background. Family type was described as single parenthood or parents who were married or cohabiting. Number of children in the family was categorized as 1 child or 2 or more children.

##### *Maternal BMI*

Mother’s weight and height was measured upon enrolment in maternity care and registered in the maternity care record as the pre-pregnancy weight. BMI was calculated as weight (kilograms) divided by height (meters) squared. The majority of the mothers (~ 90%) made their first visit after 10 weeks of pregnancy [20]. The data in the maternity care records are reported to the MBR.

##### *Maternal smoking habits 3 months before pregnancy*

At enrolment in maternity care, the women’s smoking habits 3 months before pregnancy was registered as either 1–9 or 10 or more cigarettes/day. In this study, we analysed smoking habits as yes or no.

##### *Children’s birth weight and birth length*

were available from both the MBR and from those collected from CHC. In Table 1, data from CHC were used and, when missing, the MBR data was used.

**Table 1:** Comparison of the baseline characteristics of the IDEFICS and reference populations

		**IDEFICS population**	**Reference population**	***p*-value**
		**N=1 825**	**N=1 825**	
Gender	Male	937 (51.3%)	937 (51.3%)	
	Female	888 (48.7%)	888 (48.7%)	1.000
Age (years)		6.16 (2.04)	6.16 (2.04)	0.985
Place of residence	Partille	914 (50.1%)	914 (50.1%)	
	Alingsås	333 (18.2%)	338 (18.5%)	
	Mölndal	578 (31.7%)	573 (31.4%)	
Children’s characteristics at birth	Birth weight (kg)^a^	3.53 (0.57)	3.53 (0.57)	0.693
		n = 1 742	n = 1 598	
	Birth length (cm)^a^	50.5 (2.4)	50.5 (2.4)	0.612
		n = 1 726	n = 1 586	
	Ponderal index (kg/m^3^)^a^	27.3 (2.6)	27.3 (2.7)	0.757
		n = 1 726	n = 1 586	
	Large for gestational age (LGA)^b^	50 (3.8%)	40 (2.7%)	0.113
		n = 1 316	n = 1 481	
	Small for gestational age (SGA)^b^	27 (2.1%)	33 (2.2%)	0.884
		n = 1 286	n = 1 500	

The matching between groups and children's characteristics at birth is shown. Data are presented as number and percent (%) or the mean with standard deviation (SD).

^a^ Children’s birth weights and lengths are derived from; register data, and the anthropometric data from Child Health Centres and School Health Services.

^b^ LGA, SGA variables were derived from register data.

##### *Ponderal index*

was derived from dividing birth weight in kilograms by birth length in meters cubed.

##### *Large for gestational age (LGA)*

was defined as a child born large for its gestational age: > 2 standard deviations (SD) above the mean for the Swedish gestational age and sex-specific birth weight curves [21].

##### *Small for gestational age (SGA)*

was defined as a child born small for its gestational age: < −2 SD below the mean for the Swedish gestational age and sex-specific birth weight curves [21].

##### *Twin or single birth*

Data on the numbers of infants born from each pregnancy was recorded in the delivery record.

#### Analytical and statistical methods

The children’s BMI SDS and BMI categories were calculated at 2.5, 4, 5.5, and 8 years of age for all growth data in both populations, e.g. a child included in the IDEFICS study at 2.5 years of age in 2007 also had longitudinal growth data up to 5.5 years of age in 2010 and 2011, when the data was collected.

The distribution of gender and age in the IDEFICS population was compared with the general population of each participating municipality using information from Statistics Sweden [22]. The general population statistics are updated each year on November 31. Since the children in IDEFICS were enrolled from September 2007 to May 2008, we used the data from 2007. Children in IDEFICS who were below the age of 2 years were not included in the population data of Statistics Sweden in November 2007 (n= 36), which explains why the number was 1,789 and not 1,825. A bootstrapping method was applied in order to adjust for differences in age-group proportions of relevance for the outcome of BMI SDS [23]. A thousand samples with the same age distribution as the SCB data have been drawn from the IDEFICS population with equal probability and with replacement.

Using data from Statistics Sweden in 2007, we compared educational level and median income on a household level in the three participating municipalities with the total number of 290 municipalities in Sweden [11]. Here, high educational level is >3 years education after high school including individuals with research training. Low educational level was defined as ≤ 9 years mandatory school. High and low education levels were 22% and 16% at the national level. In Partille, Mölndal, and Alingsås, the corresponding proportions were 25%, 29%, and 20% for high and 13%, 13%, and 16% for low educations levels, respectively. The national average median income was 218,000 Swedish kronor and the corresponding figures were 243,000, 244,000, and 219,000 Swedish kronor for the three participating municipalities, respectively (data not shown).

Comparisons between groups were done using the Mann–Whitney U test for continuous variables, the Mantel-Haenzel Chi Square test for ordered categorical variables, and Fishers exact test for dichotomous variables. Distributions of continuous variables are described by their means (M), standard deviations (SD), medians, and numbers (n). Categorical variables are presented as numbers and percentages. All statistical testing was 2-tailed with alpha 5%. For the study of determinants of participation in the IDEFICS study, a stepwise multiple logistic regression analysis was performed for the IDEFICS versus referent populations. Area under the curve is the AUC statistics in the ROC-curve, calculated for description of goodness of predictors. The statistical analyses were carried out with SAS statistical software package version 9.2.

### Ethics

In the European IDEFICS-project, research ethics committees in each country approved the study (for Sweden; DNR 264–07). All parents provided written consent for all examinations and/or the collection of samples, subsequent analysis, and storage of personal data and collected samples. The children were to give oral consent to the different parts of the examinations.

The present study was approved separately by the Regional Ethical Review Board at the University of Gothenburg, Sweden (DNR 089–09).

The data protection council at Statistics Sweden approved transmission of PIN´s and municipality affiliations from the IDEFICS participants and their referents to enable retrieval of anthropometric data from the children´s health care records at CHC and SHC for the comparisons of anthropometric data. However, permission to study the non-respondent group (n = 934) was not given. For the linkage of socioeconomic (SES) and sociodemographic register data, and register linkage of data from the medical birth register, an additional written informed consent from the IDEFICS participants was required by Statistics Sweden. This was not required for the referents.

## Results

A check of matching between the two populations showed good agreement with regards to the distribution of age, gender, and place of residence. The birth characteristics did not differ between the two populations (Table 1). The BMI SDS index at age of inclusion did not differ between the populations. BMI SDS at ages 2.5, 4, and 5.5 years did not differ, whereas there was a difference at 8 years of age. The mean (SD) BMI SDS of the referent and IDEFICS populations were 0.303 (1.040) and 0.191 (1.025), respectively (*p* = 0.049) (Table 2). The IOTF-BMI categories followed the same pattern, and a difference in the prevalence of obesity was present only for the 8 year old children (2.9% vs 4.5%; *p* =0.033) (Table 2).

**Table 2:** Comparison of children’s anthropometric measurements in the IDEFICS and reference populations

**Child anthropometric measurements**	**IDEFICS population**	**Reference population**	***p*-value**
	**(n=1 825)**	**(n=1 825)**	
BMI SDS index at age of inclusion^a^	0.110 (1.000)	0.169 (0.976)	0.160
	0.085 (−3.548; 4.010)	0.126 (−3.432; 3.671)	
	n = 1 644	n = 1 479	
BMI SDS at 2.5 years^b^	0.135 (0.997)	0.163 (0.984)	0.469
	n = 1 389	n = 1 265	
BMI SDS at 4 years^b^	0.063 (0.987)	0.067 (0.986)	0.978
	n = 1 622	n = 1 471	
BMI SDS at 5.5 years^b^	0.080 (1.016)	0.075 (1.009)	0.521
	n = 1 450	n = 1 333	
BMI SDS at 8 years^b^	0.191 (1.025)	0.303 (1.040)	0.049
	n = 1 060	n = 1 002	
BMI categories at 2.5 years			
Normal weight and underweight	1 227 (88.4%)	1 096 (86.6%)	
Overweight	136 (9.8%)	147 (11.6%)	
Obese	25 (1.8%)	22 (1.7%)	0.281
BMI categories at 4 years			
Normal weight and underweight	1 443 (89.0%)	1 302 (88.6%)	
Overweight	153 (9.4%)	138 (9.4%)	
Obese	26 (1.6%)	30 (2.0%)	0.577
BMI categories at 5.5 years			
Normal weight and underweight	1 272 (87.7%)	1 150 (86.3%)	
Overweight	142 (9.8%)	144 (10.8%)	
Obese	36 (2.5%)	39 (2.9%)	0.253
BMI categories at 8 years			
Normal weight and underweight	883 (83.2%)	802 (80.0%)	
Overweight	147 (13.9%)	155 (15.5%)	
Obese	31 (2.9%)	45 (4.5%)	0.033

Data are presented as means with standard deviation (SD), median (min; max), or number and percent (%).

^a^ BMI SDS index: Body mass index standard deviation score index.

^b^ BMI SDS: Body mass index standard deviation score.

The demographic characteristics of the IDEFICS population were compared with the data from the general municipality population registers [11]. There was no gender difference. For age, there was a significant difference in proportions between the populations (*p* = 0.002). The range of the differences in proportions by each 1-year age group varied from 0.2% to 3.9%, but varied in direction. The impact of the age-differences on the BMI SDS, using the bootstrapping analysis, did not show an effect on our study findings. At the ages 2.5, 4, and 5.5 years, >90% of the tests showed a non-significant result in comparison with our results. At 8 years of age, 63.9% of the tests showed that the IDEFICS adjusted population had a lower BMI SDS compared with the referents, in agreement with our findings (data not shown).

Maternal BMI at enrolment in maternity care did not differ between the two populations. The IDEFICS mothers were older than those in the referent population (37.8 [4.5] vs. 37.0 [5.2] years; *p*=0.001). No smoking 3 months before attending maternity care was reported in 86.9% versus 80.1% of mothers in the IDEFICS versus reference populations, respectively (*p*<0.001) (Table 3). The educational characteristics using the ISCED differed between the populations (*p*<0.001) (Table 3). Low level of education was infrequent in both groups, but more prevalent in the referent population, and high education level was more common among the IDEFICS parents. Several other family characteristics differed between the IDEFICS and reference populations; married or co-habiting parents vs. single parenthood, foreign vs. Swedish parental background, and the number of children in the family. In addition, family disposable income; parental personal income; social welfare-, disability- , and unemployment pension; and financial study assistance per family differed significantly. The proportion of twins in the two populations did not differ (Table 3).

**Table 3:** Register data

		**IDEFICS population**	**Reference population**	***p*-value**
		**n=1 431**	**n= 1 825**	
Maternal characteristics				
	BMI	23.8 (3.7)	24.0 (3.9)	0.479
		n = 1 158	n = 1 305	
	Age (years)	37.8 (4.5)	37.0 (5.2)	<0.001
		n = 1 370	n = 1 557	
Smoking 3 months before pregnancy	Not smoking	1 071 (86.9%)	1 095 (80.1%)	
	1–9 cigarettes/day	73 (5.9%)	133 (9.7%)	
	10 or more cigarettes/day	88 (7.1%)	139 (10.2%)	<0.001
Family characteristics				
Educational level	ISCED (1–2) Low	18 (1.3%)	49 (2.7%)	
	ISCED (3–4) Medium	492 (34.4%)	820 (45.0%)	
	ISCED (5–6) High	921 (64.4%)	953 (52.3%)	<0.001
Family type	Single parent	112 (7.8%)	269 (14.7%)	
	Married or co-habiting	1 319 (92.2%)	1 556 (85.3%)	<0.001
Number of children	One child	168 (11.7%)	287 (15.7%)	
	Two or more children	1 263 (88.3%)	1 538 (84.3%)	<0.01
Single or twin birth	Single birth	1 327 (96.9%)	1 510 (97%)	
	Twin birth	43 (3.1%)	47 (3.0%)	0.934
Parental origin	Swedish	1 337 (93.4%)	1 599 (87.6%)	
	Foreign	94 (6.6%)	226 (12.4%)	<0.001
Family economy	Disposable income/family^a^	4 943 (2 039)	4 651 (2 527)	<0.001
		n = 1 431	n = 1 825	
	Parental personal income^a^ (one parent)	2 827 (2 445)	2 078 (1 814)	0.004
		n = 112	n = 269	
	Social welfare pension/family	26 (1.8%)	85 (4.7%)	<0.001
	Disability pension/family	45 (3.1%)	103 (5.6%)	<0.001
	Unemployment compensation/family	141 (9.9%)	243 (13.3%)	0.003
	Financial study assistance/family	106 (7.4%)	194 (10.6%)	0.002

Maternal characteristics at enrollment in maternity care, and family socioeconomic and sociodemographic characteristics in the IDEFICS and reference populations. Data are presented as the mean with standard deviation (SD) or number and percentage (%).

^a^ The income is in hundred Swedish krona (SEK), e.g. 4 943= SEK 494 300 per year; this sum was equivalent to €53 449 in 2007 (€ 1 = SEK 9.2481).

The univariate logistic regression analyses (Table 4) to study determinants for participating in the IDEFICS study, were significant for clinically relevant family characteristic variables. Highest attained education high vs. low was significant (p < 0.001) but not medium vs. low (p= 0.966). The BMI SDS index remained non significant also in the univariate analysis (p= 0.219). The variables in the final model analysed in the stepwise multivariate regression analysis were parental origin, family type, and parental education. The strongest association was seen for parental origin (odds ratio [OR] = 1.91, 95% confidence interval [CI] 1.48–2.47), i.e. almost twice as many were of foreign origin in the reference group. The area under the curve for this test was 0.59 (95% CI 0.57–0.61).

**Table 4:** Multiple logistic regression analysis of odds ratios (OR) for participation in the IDEFICS study versus belonging to the reference population

		**Univariate**			**Multivariate**	
**Coefficients**	**OR**	**(95% CI)**	***p*-value**	**OR**	**(95% CI )**	***p*-value**
Family type^a^	2.04	(1.61-2.57)	<0.001	1.75	(1.38-2.23)	<0.001
Number of children in the family^b^	1.40	(1.14-1.72)	0.001			
Parental origin^c^	2.01	(1.56-2.58)	<0.001	1.91	(1.48-2.47)	<0.001
Highest attained education Medium vs Low^d^	1.63	(0.94-2.83)	0.966	1.19	(0.68-2.09)	0.436
Highest attained education High vs Low^d^	2.63	(1.52-4.54)	<0.001	1.80	(1.02-3.16)	0.001
Disposable income/family (100.000 SEK)^e^	1.06	(1.02-1.09)	0.001			
Unemployment compensation/family^f^	1.41	(1.13-1.75)	0.003			
Disability pension/family^f^	1.84	(1.29-2.63)	0.001			
Social welfare pension/family^f^	2.64	(1.69-4.12)	<0.001			
Financial study assistance/family^f^	1.49	(1.16-1.90)	0.002			
Child BMI SDS index	0.96	(0.91-1.02)	0.219			

The dependent variable is the odds of family characteristics in the IDEFICS population vs. the reference population (0 = reference population).

^a^ Reference category = Single parent.

^b^ Reference category = 1 child (1 vs. 2 or more children).

^c^ Reference category = Non-Swedish.

^d^ The ISCED educational levels are low 1–2, medium 3–4, and high 5–6.

^e^ SEK= the currency of Sweden, the Swedish krona.

^f^ Reference category = yes.

## Discussion

In this study, we found sociodemographic and socioeconomic differences between the reference and the Swedish IDEFICS populations supporting our first hypothesis. Those with disadvantageous socioeconomic and sociodemographic backgrounds were underrepresented in the population in the IDEFICS study. Our second hypothesis was not supported, since we found no selection effect related to the children’s BMI at the time and age when the IDEFICS children were included.

Our findings are supported by several other studies. Low level of education is known to have a selection impact according to several studies in adult populations [7,24]. Non-participation in a parental support program for underage drinking in adolescents was strongly associated with low education [24]. Other reported obstacles for participation are single parenthood and immigrant background, related to busy personal schedules, inconvenient times, and logistical difficulties [25]. Participation, on the other hand, was related to non-smoking habits, higher education, and co-habiting parents [26]. These associations are in line with the inverse care law, i.e. medical care tends to vary inversely with the severity of the health problem [27]. The inverse equity hypothesis is a consequence of this [28]. Accordingly, new public health interventions may increase inequity in health initially by having a stronger impact on the well-to-do families than poorer ones. However, the gap will close over time, and disadvantaged families may catch up [28].

Sweden is a reasonably homogenous society with comparatively equal income distribution [3], consequently we found no health inequity when using BMI as a health indicator [5]. Still there was a distinct unequal distribution of sociodemographic backgrounds between the two populations in our study. Also, an uneven distribution was evident despite that the three IDEFICS municipalities were largely a bit above or similar to the average national socioeconomic level. All the participants at the eight centres in the IDEFICS study were convenient samples, not nationally representative. The educational level of the various populations appeared to vary largely between the centres [29], indicating that the selection mechanisms might differ. We find it likely that a selection bias occurred in all countries but the pattern is probably unique for each one.

Our second hypothesis related to BMI was not supported. The BMI SDS Index at age of inclusion did not differ significantly between the populations. The IDEFICS study was devoted to young children aged 2–9 years. In this age group, the well-known stigma of childhood obesity may be less severe than for older school children [30]. Parent’s lack of perceiving their children’s accurate overweight or obese weight status is another possible explanation for attendance in the IDEFICS study. A previous study within IDEFICS showed that between 51% and 77% of parents to children with overweight classified their children as normal weight, and about 57% to 85% of parents of children in the obese category classified their children as “slightly too overweight” [31].

We found that the growth characteristics of the study populations at birth up to 5.5 years of age were very similar. At 8 years of age, the BMI SDS and BMI categories according to IOTF differed significantly. Growth data collected from the health care records showed that 2.9% of the IDEFICS population were in the obese category, whereas the prevalence in the referent population was 4.5% (*p* = 0.03) at 8 years of age (Table 2). Our interpretation is that the age-related development of BMI differed between the two populations. A possible explanation for this could be diverse effects on the populations over time of the “obesogenic environment” [32]. In two studies of Swedish pre-school children, the growth development was different in populations according to differences in socioeconomic characteristics [33,34]. In one of these studies, growth data did not differ by socioeconomic factors at birth, whereas children at 4 years of age in the more disadvantaged areas had a significantly higher prevalence of overweight and obesity [33].

In the present study, a selection bias in the IDEFICS population was demonstrated. Not one but several socioeconomic characteristics pointed towards a clear difference between the populations. Sociodemographic background and multiple adverse circumstances are interrelated in a complex pattern [35]. In the reference population, lower education and incomes and more financial support from society were present. Families with these characteristics may have less capacity to resist environmental influences and protect their children from them [36]. Development of obesity in children and a higher prevalence of smoking among mothers in the referent population reflect a social patterning in agreement with others [37]. Immigrant families, especially if living in a deprived area, have a higher prevalence of overweight and obesity compared with Swedish adults [38]. The reference population had a higher prevalence of immigrants in this study. This was also the strongest determinant for belonging to the reference population (Table 4), and could be an important factor in the diverse development of higher BMI at 8 years of age that was demonstrated in this study.

Considering our results, we propose the following strategies to increase representativeness in health surveys and community interventions: exploit all available socio- demographic and municipality statistics; make use of focus groups consisting of local community officials with inside knowledge of the community; to overcome culture barriers, use culture bearers, adapt and translate written and oral information to residents with foreign background and short education; single parents may benefit from flexible time-schedules in time and setting; survey and study personnel might also perform their work in the geographic vicinity of the target populations.

### Limitations

The municipalities chosen to participate in the IDEFICS study were not randomly selected, although efforts were made to choose municipalities corresponding to the average Swedish municipality. The distribution of participants was necessary to adjust to the intervention design of the IDFICS study, with recruitment of one half of the participants from one municipality and the other half from two others. Ethical approval to study the 934 non-participating families was not granted. It would have been of great value to determine the characteristics of this group. Another limitation is the relatively low AUC (0.59) of our model. However, many other circumstances that are not possible to measure and include in the model may contribute to the outcome.

### Strengths

In Sweden, the unique PIN makes it possible to link different national official register data at an individual level [12]. Using the PIN, each child in the IDEFICS study was closely matched to the referent child living in the same municipality, with only ± 1 month’s differences in age. The Swedish registers are very complete, derived directly from the authorities and have very little missing data, granting the validity of information. Further, the measured longitudinal child growth data from the health records at CHC and SHC were available for 95% of the eligible IDEFICS and 89% of the referent populations. An important strength was the unique opportunity to link the growth and register data.

## Conclusions

There was a selection bias in the IDEFICS-Sweden study, with greater participation of families with more advantageous sociodemographic backgrounds. The socioeconomic and sociodemographic differences we found were quite evident and are important to consider when interpreting survey findings. Our hypothesis that overweight or obesity in young children had an independent effect on participation in the IDEFICS survey was not supported. This has important implications for preventive interventions, suggesting that starting in early childhood seems to be beneficial. The BMI development was different in the two populations. At 8 years of age, the reference population had a significantly higher prevalence of obesity. We see this as a probable effect of environmental influences, also pointing to the value of starting prevention at an early age. We suggest that efforts in society are strengthened to give support to families characterized by single parenthood, foreign background, low education and income, in health-promoting interventions in the future.

## Competing interests

The authors declare that they have no competing interests.

## Authors’ contributions

SR was responsible for the design of the study, funding and ethical applications, growth data collection, data analysis, literature search and for drafting of the manuscript. SM, LL and GE were responsible for organizing the Swedish IDEFICS study, its design and funding. WA was PI for the entire IDEFICS Europe study. MN, GE, LL, SH and TZS participated in analysis and drafting of the manuscript. SM was in addition responsible for the design, ethical applications, data analysis and drafting of the manuscript. All authors read and approved the final manuscript.

## Pre-publication history

The pre-publication history for this paper can be accessed here:

http://www.biomedcentral.com/1471-2458/13/418/prepub
